# Demand versus supply in intensive care: an ever-growing problem

**DOI:** 10.1186/cc13199

**Published:** 2014-03-17

**Authors:** M Tanaka Gutiez, R Ramaiah

**Affiliations:** 1 East Kent Hospitals University NHS Foundation Trust, Ashford, UK

## Introduction

The demand for intensive care has reached new heights, where medical advancement and aging populations have increased the proportion of patients with multi comorbidities. Ideally, bed occupancy on the ICU should be around 70% whereas beyond 80% has been associated with increased mortality. William Harvey Hospital is a district general hospital with nine ICU beds where the ICNARC national database suggested a consistently high occupancy. The purpose of this study was to assess bed occupancy and its impact on service delivery.

## Methods

Data were collected between 2005 and 2013 to analyse trends in ICU bed occupancy, overnight discharges, surgical cancellations, and nonclinical transfers. Data were gathered from April 2012 to March 2013 using quality indicators (Intensive Care Society): readmissions; premature discharges; discharges at night; cancelled planned surgery; and nonclinical transfers. Regression analysis was conducted to assess: correlation between mortality and effects of excess occupancy; and causality of increasing demand for ICU beds.

## Results

Bed occupancy remained high between 2005 and 2013 with a 15% increase. This was associated with increased overnight discharges (29%), surgical cancellations (90%), and nonclinical transfers (88%). Between 2012 and 2013, the calculated demand for beds was 174. There were 1.8 ICU to 100 hospital beds and 4.5 ICU beds per 100,000 population, which dropped significantly when including regional specialty services. A persistent gap between ICU bed supply and demand was associated with increased unfavourable outcomes in all quality indicators.

## Conclusion

A steady increase in occupancy over the last 8 years was due to multiple factors, including an increase in clinical services such as in the coronary care centre, head and neck unit, and trauma centre. Presentation of our results to managerial level facilitated increased bed capacity by 22%.
